# First Observation of Tool Use in Wild Gorillas

**DOI:** 10.1371/journal.pbio.0030380

**Published:** 2005-10-01

**Authors:** Thomas Breuer, Mireille Ndoundou-Hockemba, Vicki Fishlock

**Affiliations:** **1** Wildlife Conservation Society, Mbeli Bai Study, Nouabalé-Ndoki Project, Brazzaville, Republic of Congo,; **2** Max Planck Institute for Evolutionary Anthropology, Leipzig, Germany; Emory UniversityUnited States of America

## Abstract

Descriptions of novel tool use by great apes in response to different circumstances aids us in understanding the factors favoring the evolution of tool use in humans. This paper documents what we believe to be the first two observations of tool use in wild western gorillas *(Gorilla gorilla)*. We first observed an adult female gorilla using a branch as a walking stick to test water deepness and to aid in her attempt to cross a pool of water at Mbeli Bai, a swampy forest clearing in northern Congo. In the second case we saw another adult female using a detached trunk from a small shrub as a stabilizer during food processing. She then used the trunk as a self-made bridge to cross a deep patch of swamp. In contrast to information from other great apes, which mostly show tool use in the context of food extraction, our observations show that in gorillas other factors such as habitat type can stimulate the use of tools.

## Abstract 2

The first documentation of tool use by wild western gorillas

## Introduction

Tool use is defined as “the employment of an unattached environmental object to alter more efficiently the form, position, or condition of another object, another organism, or the user itself when the user holds or carries the tool during or just prior to use and is responsible for the proper and effective orientation of the tool” (p. 10 of [1]). Beck's classic book [1] defines six different types of tools: objects thrown at predators or rivals, objects used to hit predators, hunting weapons (only hominids), objects incorporated into social displays, objects to clean body parts, and objects made and used to acquire food, such as insects or nuts [1,2].

Information on tool use and factors favoring tool use in wild apes helps us to understand its importance in the evolution of our own species. Although there are reports of tool use by captive gorillas (*Gorilla* sp.), including object throwing and use of tools in feeding [3–9], there has been to our knowledge no reported case of tool use in by wild gorillas, despite decades of field research. It has been argued that gorilla tool use in captivity is less extensive than that shown by other apes [10], but it has recently been demonstrated that captive western gorillas *(Gorilla gorilla)* showed tool-using skills similar to those of orangutans *(Pongo pygmaeus)* [11], who frequently use tools in the wild [12].

One possible explanation for the absence of observed tool use in wild gorillas is that they are less dependent on extractive foraging techniques that might require the use of tools, since they exploit food resources differently than chimpanzees *(Pan troglodytes)* [2,13]. Whereas chimpanzee feeding ecology involves tools such as hammers to crack open nuts and sticks to fish for termites, gorillas access these food resources by breaking nuts with their teeth and smashing termite mounds with their hands. Nevertheless, mountain gorillas *(Gorilla beringei)* possess food-processing skills of comparable complexity and logical organization to chimpanzee termite fishing [14], which give them access to additional herbs in their habitat [15].

Here we report two instances of novel tool use by gorillas, both involving using detached branches to give postural support to cross water and swamp at Mbeli Bai, a forest clearing in northern Congo.

## Results

On 9 October 2004, we observed George's group (monitored since 1995) near the edge of the clearing 180 m from the observation platform. This group had not been observed to use this area for more than 6 mo, and meanwhile elephants had created a new pool to feed on soil. Adult female Leah was seen at the pool edge near where a branch was sticking out of the surface, looking intently at the water in front of her for 1 min before she entering the water (Figure 1, top left). She began to cross the pool walking bipedally, but after her first steps the water quickly became waist deep and she returned to the pool edge. Leah then re-entered again bipedally and grabbed the straight branch in front of her with her right hand (Figure 1, top center). Relative to Leah's body size we estimated the leafless branch as being approximately 1 m long and 2 cm thick. Leah then detached the stick and, stretching forward with it in her right hand, seemed to use it to test the water depth or substrate stability: she grasped the stick firmly and repeatedly prodded the water in front of her with the end of the stick (Figure 1, top right). She then moved further into the pool, holding the detached branch in her right hand and using it as a walking stick for postural support (Figure 1, bottom three images). She advanced a further 8–10 m from the pool edge (not shown), repeating the actions shown in Figure 1, and then, leaving the stick in the pond, returned to her entry point, where her offspring was crying. She did not re-enter the water, but instead moved around the pool to feed on aquatic herbs.

**Figure 1:**
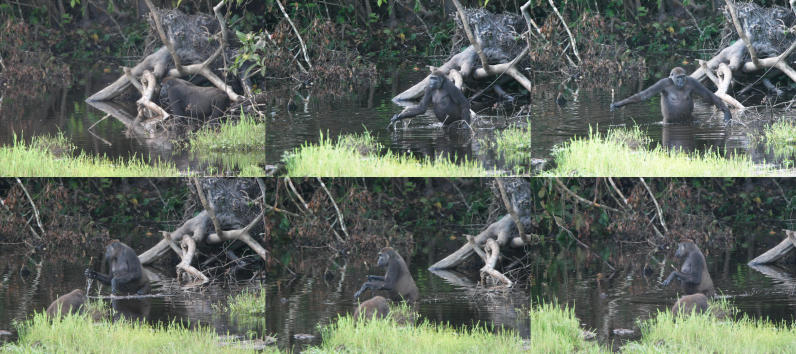
Female Leah Using a Walking Stick while Crossing Bipedally through an Elephant Pool at Mbeli Bai Female Leah first looked at the new elephant pool and the branch she later used as the walking stick, and entered the water without the tool (not shown). After re-entering the pool and taking the branch with her right hand, she walked bipedally 8–10 m into the water, frequently testing water deepness.

The second observation involved female Efi from Zulu group (monitored since 2000), who entered the clearing on 21 November 2004, 150 m from the observation platform. While close to the forest edge Efi detached a 1.3-m-long and 5-cm-thick leafless trunk of a dead shrub with both hands. She forcefully pushed it into the ground with both hands (Figure 2, middle) and held the tool for support with her left hand over her head for 2 min while dredging food with the other hand (Figure 2, bottom). Efi then took the trunk with both hands and placed it on the swampy ground in front of her, crossed bipedally on this self-made bridge, and walked quadrupedally towards the middle of the clearing (not shown). We could not see if the bridge was long enough to cross the swampy ground, but it certainly gave the female more stability underfoot and supported her weight for at least part of the distance.

**Figure 2:**
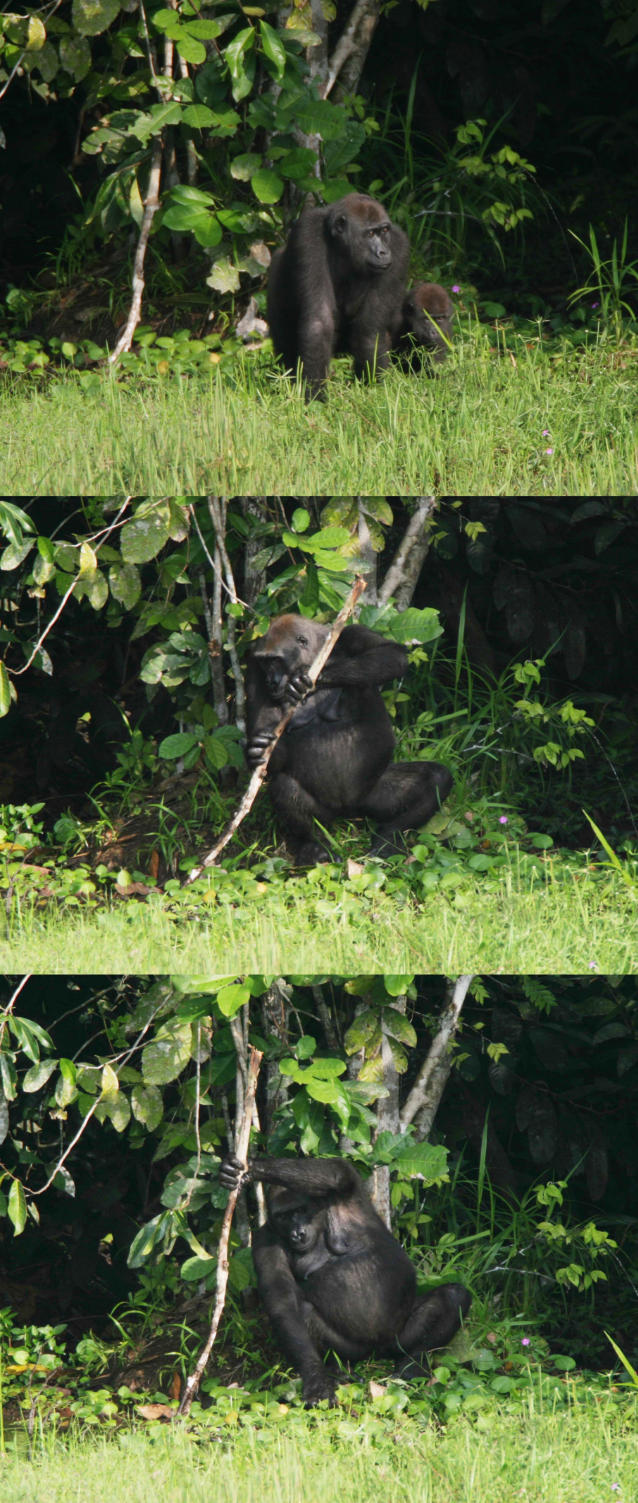
Female Efi Using Trunk as a Stabilizer during Food Processing at Mbeli Bai The top photo shows the intact trunk shortly before Efi manipulated it (visible to the left of female Fulani). The trunk was then detached by female Efi with both hands (middle), pushed into the ground, and used as a stabilizing stick while dredging aquatic herbs towards her with her other hand (bottom).

## Discussion

It has been proposed that animals that can obtain resources by force may not need to rely on tools [2,13], but our observations show that other functional demands may also stimulate tool use, at least in apes. Our observations of gorilla tool use for postural support in a swampy habitat supports arguments that the use of tools reflects ecological needs and may therefore be viewed as an adaptation to a particular suite of environmental conditions [14]. Swamp habitat demands particular behavioral adaptations for western gorillas and may stimulate special behaviors, such as the splash display of adult male silverbacks at Mbeli Bai [16]. The forest edges of Mbeli Bai are heavily inundated, and gorillas often use branches to haul themselves out of the water or move around the edge of the clearing, but the cases reported here are the first observations, to our knowledge, of wild gorillas using detached objects as tools.

The observed tool use involved gorillas from two different groups and thus could indicate independent inventions, perhaps reflecting past negative experiences with deep water. However, there is also the intriguing possibility that using branches to test water depth or as bridges is a more common adaptation to this particular habitat. Footprints of gorillas walking over branches as bridges have been observed in other swampy clearings not far from Mbeli Bai [17].

We make no hypothesis about the mode of acquisition of this tool use behavior, but simply note that the high population density of western gorillas in northern Congo [18–20] makes social learning a possible transmission route [2].

All great apes use tools in captivity, but until recently tool use in the wild was only regularly observed in chimpanzees and orangutans [12,21,22]. There are now reports of tool use by bonobos *(P. paniscus)* [23] and western gorillas, which highlights the importance of long-term studies in these species.

## Materials and Methods

Mbeli Bai is a 12.9-ha large swampy forest clearing in the in the southwest part of Nouabalé-Ndoki National Park, Republic of Congo. The clearing includes streams and elephant pools and is dominated by aquatic vegetation in the Cyperaceae, Gramineae, and Hydrocharitaceae families. The aquatic vegetation forms a floating surface to the bai, with an average depth of 1 m [24]. Monitoring of the social organization, demography, and behavior of western gorillas at Mbeli Bai has been ongoing since 1995 [25,26]. Observations are made with 15–45 × 60 mm telescopes from a 9-m-high viewing platform overlooking the clearing. Gorillas are habituated to the presence of researchers and can be identified by their distinctive features, such as pelage, nose prints, and shape of brow ridges [25].
